# Supplementary material for: Pharmacologic activation of Δ133p53α reduces cellular senescence in progeria patients-derived cells
Source: bioRxiv. 2025 Aug 2:2025.07.28.667224. Originally published 2025 Jul 31. Preprint. [Version 2] doi: 10.1101/2025.07.28.667224 (PMC12324402; doi:10.1101/2025.07.28.667224)
Supplement: Supplement 1 [file media-1.pdf]

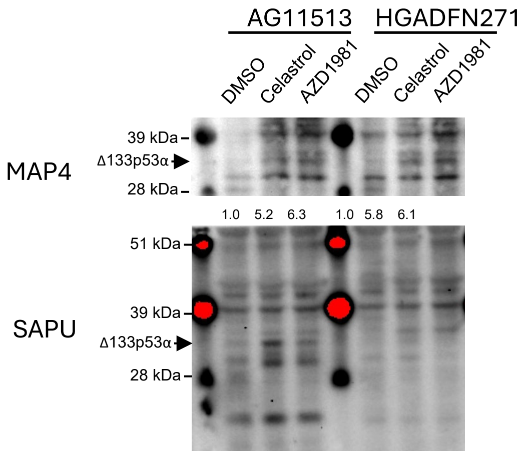

Supplementary Figure 1. Original western blot images obtained with MAP4 and SAPU antibodies. The images obtained with the rabbit polyclonal antibody MAP4 (above; cropped bands shown in Figure 2) and the sheep polyclonal antibody SAPU (below) both consistently showed bands corresponding to Δ133p53α, which were upregulated by celastrol and AZD1981 (indicated by arrows).

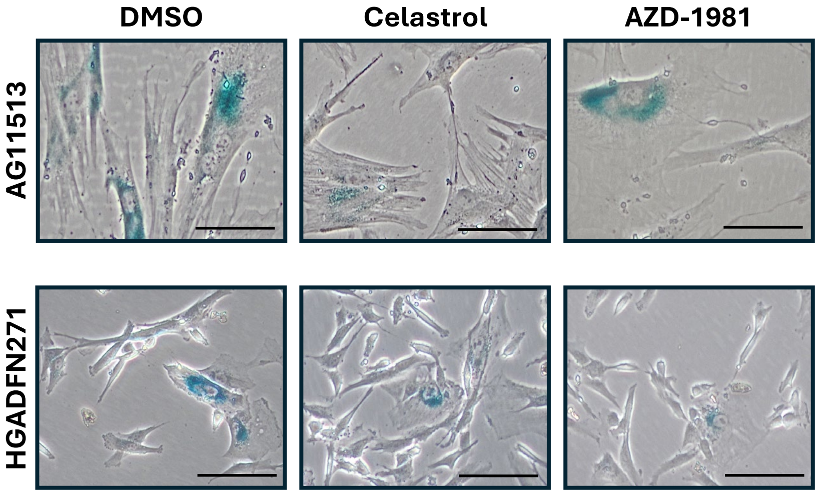

Supplementary Figure 2. Representative images of SA-β-gal staining. Magnification, ×40. Scale bars, 50 µm.
